# ACTION for Men: Study Protocol of a Community Capacity Building Intervention to Develop and Implement Gender-Sensitive Physical Activity Programs for Men 50 Plus

**DOI:** 10.3389/fpubh.2020.00004

**Published:** 2020-01-24

**Authors:** Helmut Strobl, Nicola Brew-Sam, Janina Curbach, Boris Metz, Susanne Tittlbach, Julika Loss

**Affiliations:** ^1^Instiute of Sports Science, University of Bayreuth, Bayreuth, Germany; ^2^Department for Epidemiology and Preventive Medicine, University of Regensburg, Regensburg, Germany

**Keywords:** health promotion, male, gender, participatory approach, setting

## Abstract

**Introduction:** Capacity building for health promotion is a relevant precondition for sustainable, health-related changes in community settings. So far, there are few evidence-based recommendations about how to implement and evaluate community capacity building approaches. ACTION for men (A4M) is a project designed to build and evaluate capacities for health promotion in three rural communities in Bavaria, Germany, via a participatory approach including multiple community stakeholders. The project specifically aims at improving physical activity (PA) in men over 50 years of age (50 plus).

**Methods and Analysis:** As a strategy to build the communities' capacities, we set up stakeholder groups in so far two communities. Those stakeholder groups will be facilitated over a period of 1–3 years. In regular meetings, the group members will be motivated to actively participate in planning and implementing PA programs for men 50 plus. The facilitation will systematically address key domains of community capacity (e.g., critical awareness, problem assessment, resource mobilization). The evaluation of the capacity building processes will be carried out using a mixed-methods design. Evaluation instruments consist of structured documentations and face-to-face interviews with stakeholder group participants (and drop-outs) as well as a pre-post-test using a standardized questionnaire in order to detect activity-related changes in men 50 plus from the involved communities. In community three, we will conduct the same procedure with a delay of 6 months.

**Discussion:** Building community capacity for health promotion programs is the primary aim in A4M, and thus differs from previous research in which capacity is mostly a means to an end or an “incidental” result of a health promotion program. Therefore, A4M is expected to deliver important findings about how to implement and evaluate capacity building processes for health promotion, as well as how to address physical activity in community settings.

## Introduction

Benefits of regular physical activity (PA) for different population groups are well-established. Among middle-aged, older and elderly adults, being physically active has been shown to improve overall health as well as mental health and to prevent chronic diseases; it is also associated with longer life expectancy (1). As prevalence of insufficient PA is high, especially in Western high-income countries (2), interventions and programs promoting health enhancing PA have the potential to positively influence public health outcomes. However, researchers have shown that men over 50 years of age (50 plus) are underrepresented in those programs (3, 4). Obviously, those programs often hold little manly appeal and consequently fail to influence many men's self-health practices (5). This is of particular concern, as men, compared to their female counterparts, are more likely to have a shorter life expectancy and experience higher mortality rates associated with chronic diseases (6). Thus, men 50 plus can be described as an important, yet hard-to-reach target group for health promotion.

To promote health enhancing behaviors in hard-to-reach target groups, *setting approaches* were previously recommended (7). A setting is a certain environment in which people live, learn, work or spend their spare time, such as school, workplace, or community (8). Municipal living environments (city or community) represent particularly promising settings for health promotion (9). Referred to as “supersettings” (9), they offer the possibility to support and to coordinate health-promoting activities of diverse organizations and groups of the respective communities. As a key principle of setting approaches, participatory interventions have been proven successful to implement health enhancing measures (7). They include a collaboration of relevant members of the respective settings in order to plan, implement, and evaluate actions promoting health enhancing behaviors such as PA in the population (10). This procedure enables the use of existing resources and the creation of necessary skills and structures for sustainable health-promoting action within the population, also described as capacity building (11, 12).

Capacity building is regarded as a fundamental prerequisite for health-related changes and for a sustainable impact on public health (13–18). As a core principle of health promotion worldwide it is a required action in the WHO Bangkok Charter for Health Promotion (12). It is necessary to support effective health promotion practice by the advancement of knowledge and skills among practitioners, the expansion of support and infrastructure for health promotion in organizations, and the development of cohesiveness and partnerships for health in the community (19). Capacity Building approaches have been successfully applied in various fields of health promotion (20–23). In community settings, the concept is also known as “community capacity” (11, 24). Community capacity aims at improved organization and mobilization of community members (individuals and organizations) for taking active and self-responsible control over health-related processes. Thus, the concept resembles community development or community empowerment approaches (25, 26).

The central dimensions characterizing community capacity largely overlap in the (health promotion) literature (11, 15, 16, 24, 26). As part of what Liberato et al. (11) call the “Hawe model,” the five dimensions organizational development, workforce development, resource allocation, partnerships and leadership were proposed (15, 16). Gibbon et al. (24), Laverack and Wallerstein (26), and Laverack et al. (27) specified these dimensions further, describing the following nine dimensions: participation (the active involvement of relevant stakeholders of the community); leadership qualities (to enable groups working efficiently); organizational structures (to enable participatory discussions); awareness of problems (critical reflection of the status quo in the community); problem analysis and problem solving (development of solutions for existing problems); resource mobilization (mobilization of financial, material and personal resources); networking with other actors (to implement worked out solutions); relationship with clients and experts (assumption of responsibility for the intervention in the long-term); and program implementation (clear defined roles and responsibilities to control the implementation of planned measures).

To facilitate a capacity building process, bringing together experts (e.g., scientists) and various relevant stakeholders within a setting (e.g., representatives of municipal administration, health promotion professionals, and citizens, for example belonging to specified populations of interest) is a necessary precondition (28–30). The corresponding communication and interaction processes can enhance knowledge and competencies concerning evidence-based and feasible health promotion interventions among all involved groups and individuals (31). Furthermore, this can enable efficient organization and mobilization of groups, resulting in an overall problem-solving capacity (i.e., the ability of the group to deal with new problems and respond to unfamiliar situations) (15).

Despite the relevance of capacity building processes for health promotion, there is little published research about how to build community capacity systematically with regard to the design and implementation of PA programs for specific populations of interest. For this reason, ACTION for men (A4M) is designed to build and evaluate capacities for physical activity programs in three rural communities, using a participatory, multi-stakeholder approach. The project is part of a transdisciplinary research network *Capital4Health* (https://www.capital4health.de), which aims at fostering health enhancing PA in various population segments and settings (30). A4M specifically targets men 50 plus, as a variety of PA programs have shown potential to increase PA in men if they are gender-sensitive and address a socio-culturally shaped understanding of masculinity (32). This study protocol illustrates the detailed research procedure we propose to apply in A4M. We indicate how community stakeholder groups can be systematically set up as a strategy to build capacity in the community setting. We suggest how these groups can be guided in order to develop and implement gender-sensitive physical activity interventions addressing men 50 plus. Furthermore, we introduce the steps for evaluating the capacity building process.

## Methods and Analysis

### Study Design

**Figure 1:**
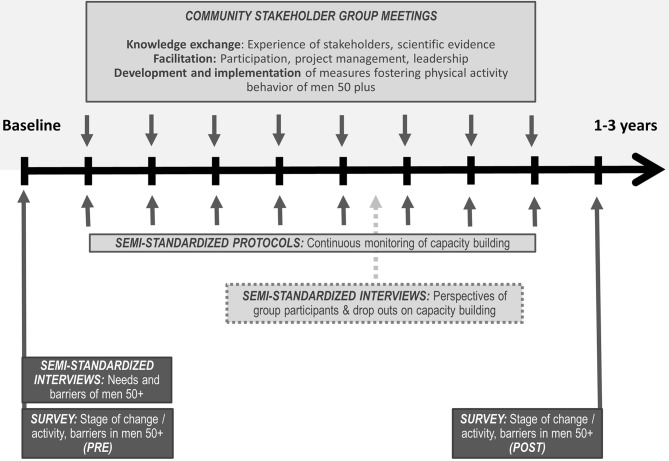
Study design of ACTION for men.

During the writing and submission process of this paper, some of the outlined activities already started (baseline assessment and recruitment of community stakeholder group). Therefore, we indicate in the following what activities have been initiated since we began preparing this manuscript and what activities we are still planning to do. So far, A4M is implemented in two rural communities (10,000–20,000 inhabitants each) in a socio-economically relatively disadvantaged county of Bavaria in Germany. Health data show an increased mortality of men in this region (33). In a third community from the same region, A4M will be implemented with a delay of 6 months to draw on the experience of the conducted activities in the other two communities. Figure 1 provides an overview of the study design and the process of the project as described in the following. Ethical approval for A4M was obtained by the Research Ethics Committee of the University of Bayreuth.

#### Baseline Assessment—Psychosocial Correlates of PA

To gain insights into relevant psychosocial correlates of PA (34) in men 50 plus, a standardized paper-and-pencil survey, supplemented by semi-structured interviews, was conducted with men 50 plus residing in the two communities involved at baseline (mixed-methods approach; further information about the recruitment process is presented in the next section Recruitment of baseline assessment participants). The survey included questions on self-reported PA in men 50 plus and selected psychosocial correlates of behavior change (for a detailed description see Table 1). Sociodemographic variables were collected by questionnaire including age, marital status, occupation and educational level. In addition, participants were asked to provide information about specific needs regarding PA and their satisfaction with existing PA offers in their communities. The supplementary semi-structured interviews with selected men 50 plus from the communities followed an interview guide with open-ended questions. The interview guide reverberated the aspects included in the paper-and-pencil survey, but was flexible to probe some aspects further depending of the results of first pilot interviews. After obtaining informed consent from the participants, the interviews were audio recorded. The interviews took place in a setting accessible for the participants and lasted between 45 and 60 min. The data from the survey and the interviews has not yet been evaluated (further information about the data analysis strategy is presented in section Data Analysis).

**Table 1:** Overview of questionnaire components and measurement details.

**Outcome**	**Equipment**	**Description**
**SELF-REPORTED PHYSICAL ACTIVITY**		
Total energy consumption	FINNISH GERMAN study on physical activity, fitness and health questionnaire (35, 36)	Participants recall information on type of activity, duration and intensity Guidelines for data processing and score creation are provided
**PSYCHOSOCIAL CORRELATES OF BEHAVIOR CHANGE**		
Intention to engage in PA	1 item assessing intention to engage in PA (37)	Participants are asked to reflect their strength of intention to engage in PA regularly in the next four weeks Response format is on a six-point scale ranging from 0 (“I don't have this intention at all”) to 5 (“I have a strong intention”)
Action self-efficacy	1 item assessing confidence in one's capacity to perform a behavior (37)	Participants are asked to reflect their capability of engaging in PA regularly within the next 4 weeks Response format is on a six-point scale ranging from 0 (“I am not confident at all”) to 5 (“I am totally confident”)
Perceived barriers to PA	15 items assessing perceived reasons hindering engagement in PA (38)	Participants are asked why PA could not be conducted regularly Response format is on a seven-point scale ranging from 0 (“I don't agree at all”) to 6 (“I totally agree”) A summary score is calculated to provide a measure for four factors (lack of time, lack of motivation, lack of self-efficacy, lack of social support) where lower scores indicate lower perceived barriers
Outcome expectations	18 items assessing expected benefits of PA in the long- or short term (38)	Participants are asked which benefits they expect from engaging in PA Response format is on a seven-point scale ranging from 0 (“I don't agree at all”) to 6 (“I totally agree”) A summary score is calculated to provide a measure for four factors (health and fitness, body and shape, performance, sociability) where higher scores indicate higher expected benefits
Stages of change	Six statements assessing the current stage of PA (34, 38, 39)	Participants are asked if they engage in at least moderate-intensive PA for an accumulated time of at least 150 min per week Response format is on six statements ranging from 1 (“No, within the last year I was not and I am not thinking about starting in the future”) to 6 (“Yes, I did engage in physical activity as such, for 12 months or more”)
Capabilities	Three items assessing individuals' self-reported capabilities (40)	Participants are asked to reflect their opportunities to achieve certain goals and to live a life that they have reason to value Response format is on a seven-point scale ranging from 0 (“Very bad”) to 6 (“Very good”)

#### Recruitment of Baseline Assessment Participants

Participants for the baseline interviews were recruited through purposeful sampling (41). The selection of participants was based on a prior definition of participants' characteristics, i.e., an equal distribution of physically active and inactive men 50 plus. To determine activity level, interested participants had to complete the self-reported PA questionnaire (see Table 1). Participants were then divided into meeting or not meeting the recommended physical activity guidelines. All attendees were offered a financial incentive of 30 € for taking part in the interview. To recruit participants for the baseline survey, all men 50 years and older living in the two communities currently involved received a written exemplar of the questionnaire with a postage paid return envelope. Addresses of men were selected based on the demographic variable “age” by an employee of the resident's registration office and provided to the researchers according to the current privacy policy. Three weeks after sending the questionnaires to the men, a reminder letter was sent to all of them, regardless of whether they participated in the survey or not. All study participants were asked for informed consent prior to participation in included data collection methods.

#### Recruitment of Community Stakeholder Group

Participants for the community stakeholder groups in the two communities currently involved were selected by means of opportunistic sampling (41). In a first step, the research team presented the project idea to the mayor as well as to the municipal council of the respective communities. Subsequently, supported by the local political representatives, a kick-off event served to find interested persons with a relevant background willing to participate in the community stakeholder groups. Special attention was paid to the fact that representatives of the population of interest were also part of the stakeholder groups.

#### Systematic Development of Community Capacity Using Stakeholder Groups

Following the baseline assessment, A4M focuses on nine capacity dimensions from the literature (11, 15, 16, 24, 26, 27) to build community capacities for the development and implementation of measures to promote PA in men 50 plus (for the dimensions see Table 2). According to the systematic of Ubert et al. (42) the capacity building strategy contains elements of community-based coalition and network building as well as professional training in institutions and organizations and training of laypersons.

**Table 2:** Dimensions of capacity building (11, 15, 16, 24, 26) and their implementation in the project.

**Dimension**	**Project implementation**
1. Participation (active involvement)	The goal is a broad representation of relevant stakeholders in the community environment (politics, business, associations/organizations relevant for health promotion, target group, interested persons) in the community stakeholder group. In the regular meetings, the research team ensures equal participation of all actors in discussions and decision-making.
2. Leadership qualities	At the beginning of the planning process, the research team takes on a directive role by clearly defining objectives and structuring and distributing tasks. In the further course of the project, the aim is to pass on this role to formal (e.g., mayor) or informal leaders from the community stakeholder group.
3. Organizational structures	Through the formation of community stakeholder groups, organizational structures are created so that citizens can network and discuss their health-related concerns and problems.
4. Awareness of problems	Stakeholder group members are encouraged not to simply accept existing circumstances in the community and to regularly reflect on and question important decisions of the stakeholder group.
5. Problem analysis and problem solving	By critically reflecting on the status quo of PA prerequisites in the community and by exchange of evidence-based knowledge about gender-sensitive PA promotion as well as professional and non-professional knowledge of group members, the stakeholder groups should be enabled to identify and solve problems.
6. Resource mobilization	As needed, stakeholder group members are encouraged to acquire financial, material and/or personal resources. The use of the resources made available is decided jointly.
7. Networking with other actors	As needed, stakeholder group members are encouraged to establish contacts with other individuals and organizations who can help to implement the proposed solutions.
8. Relationship with clients and experts	At the beginning, the research team plays a leading role in initiating the project. Gradually, however, the researchers only moderate the planning process; the decision-making power and authority lies within the stakeholder groups. At the end of the process, the research team can withdraw.
9. Program implementation	The research team works toward communicating aspects of project management to the stakeholder groups. Gradually, together with the stakeholder groups, the responsibilities for specific work steps, and the coordination of the process are to be distributed to stakeholder group members so that the groups are able to plan and implement measures to promote PA independently.

Initially, community stakeholder groups in the two communities currently involved were established consisting of (1) members of the research team as well as partners from local (2) politics, (3) business, (4) associations/organizations relevant for physical activity, (5) associations/organizations relevant for (older) men, and (6) the health care system. Furthermore, (7) men 50 plus as well as (8) other interested citizens participate in the stakeholder groups (for further information about the recruitment process see the previous section Recruitment of community stakeholder group). Regular group meetings will be facilitated from now on over a period of 1–3 years. The research team will ensure equal participation of all stakeholders in discussions and decision-making (capacity dimensions 1 and 3, Table 2).

To enable efficient organization and mobilization of the group, the research team will strive to provide a clear project management structure. This implies the moderation of the discussions and decision-making processes, fostering the definition of objectives as well as structuring and distributing tasks. Gradually, a formal (e.g., mayor or project coordinator) or informal leader from the community stakeholder group is assumed to take over this role. Thus, the research team is planned to withdraw in the course of the project (capacity dimensions 2, 8 and 9, Table 2).

To enhance knowledge and competencies concerning evidence-based and feasible health promotion interventions, the research team will systematically foster knowledge exchange within the stakeholder groups. Scientific evidence will be provided by the research team regarding gender-sensitive promotion of PA in men 50 plus based on systematic literature reviews on PA programs especially designed for men (43). Further information about barriers, attitudes, and needs toward PA of men 50 plus will be drawn from the baseline standardized paper-and-pencil survey and the semi-structured interviews with men 50 plus in the respective communities. Additionally, the research team will invite the stakeholder group participants to contribute their own professional and non-professional knowledge, including individual perceptions and experiences regarding existing health promotion programs in the community (e.g., existing PA programs for men). The overall aim is to reflect critically the status quo of PA prerequisites in the respective communities and to find solutions for gender-sensitive PA promotion (capacity dimensions 4 and 5, Table 2). Moreover, stakeholder group participants will be encouraged to establish contacts with other individuals and organizations who can help to implement the proposed solutions and to acquire personal or financial resources (capacity dimension 6 and 7, Table 2).

#### Procedure for the Single Stakeholder Group Meetings

Based on experiences and recommendations from other projects (28, 29, 44, 45), the aim is to guide the community stakeholder groups through different phases, thus both guaranteeing participation, building their capacities, and making sure that PA interventions will be implemented. The first step is a brainstorming of ideas for activities within the group, which will afterwards be structured and clustered by the research team. The next meetings will serve to discuss, change and prioritize ideas and then to agree upon results. In the following meetings, the groups will focus on the development of planned measures, including assigning specific steps to be taken for each measure, developing a time schedule for implementation, clarifying responsibilities for different implementation tasks, addressing resources needed and allocated and determining indicators of successful implementation. The duration of each of these phases (from one to several meetings) may vary between communities. The research team will accompany each stakeholder group until PA programs are implemented and evaluated.

### Outcome Assessment

#### Psychosocial Correlates of Physical Activity

To detect community-specific changes in the selected psychosocial correlates of PA (see Table 1) due to the developed and implemented PA programs by the respective stakeholder groups (see section Procedure for the single stakeholder group meetings), a post-evaluation using the same standardized paper-and-pencil survey as used at baseline will be conducted. The questionnaire will be sent to the same addresses as used for the baseline assessment after finishing the implementation process of PA measures, following the same procedure as applied at baseline.

#### Evaluation of the Capacity Building Process

In order to evaluate the capacity building process, structured documentation of stakeholder group meetings, semi-structured interviews with stakeholder group participants (and drop-outs) as well as a structured documentation of PA measures will be used.

A structured documentation of the discussions as well as the events in the individual stakeholder group meetings will be carried out. For this purpose, two researchers will regularly take part in the stakeholder group meetings and will note respective events, interactions, decisions, and procedures during and after the meeting (minutes). Subsequently, the events in the stakeholder group meetings will be discussed against the theoretical background of the capacity dimensions (e.g., critical awareness of the participants, development of leadership quality) by the two researchers, and minutes will be completed in accordance with the dimensions of the capacity development (see Table 2) in consensus. Through the repeated preparation of these minutes after each stakeholder group meeting, dynamical changes in the capacity characteristics can be recorded.

Semi-structured interviews will be conducted with stakeholder group participants, following an interview guide with open-ended questions. The interview guide will be based on the dimensions of capacity building, specified for physical activity (e.g., asking for the perceived development of critical awareness or resource mobilization in the group). In addition, the interviewee's motivation for the participation in the stakeholder group, his or her expectations and potential benefits, as well as reasons for maintaining commitment (or for dropping out) will be addressed. After obtaining informed consent from the participants, all interviews will be audio recorded. The interviews will take place in a setting accessible for the stakeholders and will approximately last between 45 and 60 min. If participants leave the stakeholder group in the course of the project, interviews will also be conducted with these dropped-out participants. The interviews will especially focus on stakeholders' experiences with the capacity development process.

The activities initiated by the community stakeholder group and their implementation at community level will be systematically documented and analyzed. Therefore, the researchers will record the number as well as the type of new activity related programs and will examine their usage by men 50 plus (number and age of participating men). For selected programs, short questionnaires will be delivered to male participants. Questions will explore reasons for joining, usefulness of program components, experience with the program and coaches, and any suggestions for improvement. Finally, two researchers will discuss and assess the implemented programs against the theoretical background of gender-sensitive activity related programs provided in the meetings of the stakeholder group. Disagreements will be resolved through discussion.

### Data Analysis

#### Interviews With Stakeholder Group Members and Documentation of Group Meetings

Standards for qualitative research by Mays and Pope (46) will guide the qualitative data analysis process. Interviews will be transcribed verbatim. Data obtained from interviews and documentations will be analyzed via thematic analysis (47). In thematic analysis, themes or patterns are searched for across a data set. Therefore, two researchers will independently analyze the transcripts. The coding frame will be based on the dimensions of capacity building but will also allow unanticipated themes to emerge and be systematically explored. Coding will be conducted by ATLAS.ti Version 7 software. The overall process will be dynamic and iterative, and several researchers will discuss the results to ensure robust and consistent analysis. Deviant cases and contradictory data will be analyzed with particular attention and discussed within the research team (47). Triangulation of data of the different data sources will strengthen the credibility and confirmability of the study results (48).

#### Standardized Survey Among Men 50 Plus

Regarding quantitative data, all variables will be checked for plausibility and missing values. Internal consistency of the instruments used will be assessed by calculating Cronbach's Alpha. Observed values at baseline and post-test will be summarized descriptively for all outcome assessments. Prior to inferential analysis, data will be checked for normality to ensure usability of parametric statistics. If applicable, hierarchical cluster analysis in combination with discriminant analysis will help to identify relevant subgroups of men 50 plus and their particular characteristics to address PA promotion measures specifically to those subgroups. General linear models for repeated measurements will serve to examine changes in outcomes from baseline to post-test. Residuals from models will be explored and will be subject to assumptions checks. Interpretation of study results will primarily be based on estimation and associated 95% confidence intervals. Two-sided *p* < 0.05 will be reported as statistically significant. Analyses will be performed using SPSS version 26.

#### Interviews With Men 50 Plus

Qualitative data from interviews with men 50 plus augment the quantitative analysis. The coding frame for thematic analysis will be based on the selected psychosocial correlates of behavior change (see Table 1). Coding will be conducted by ATLAS.ti Version 7 software. Focus of analysis will be both on unique as well as confirmatory elements.

## Discussion

In two German communities, we initiated a group of community representatives and stakeholders which is intended to develop and implement local physical activity interventions aimed at men 50 plus. In a third community, we will conduct the same procedure with a delay of 6 months. By this participatory approach, we intend to make the involved communities responsible for, and more capable of, conducting gender-sensitive physical activity programs and maintaining those programs (16). We propose to evaluate this capacity building process using a mixed-methods approach. The main focus of the evaluation will be to analyze how the nine capacity dimensions displayed in Table 2 (11, 15, 16, 24, 26, 27) within the respective community groups develop over time. We will employ semi-standardized documentation of group meetings, semi-standardized interviews with current as well as dropped-out members of the community stakeholder groups, and tracking of local interventions and changes with regard to physical activity. This evaluation is framed by a pre-post-survey among men 50+ in the respective communities, covering, among others, their physical activity behavior, barriers and facilitators as well as awareness of local resources and programs. The results of the baseline survey, alongside the results of a qualitative interview study on PA barriers and facilitators and a systematic review on male-specific PA programs, will be presented to the respective stakeholder group in order to inform the planning process and build capacities (e.g., critical awareness, problem solving).

### Strengths and Limitations

There is no gold standard for evaluating the processes and outcomes of capacity building in communities, especially for the field of physical activity promotion. In a recent literature review of community capacity building for physical activity promotion among older adults, Ubert et al. (42) found that none of the included publications explicitly assessed capacity building as an outcome. The authors stated that some research groups summarized their “lessons learned” in terms of facilitators and barriers in the capacity building process. We intend to gather information on capacity building in a more structured way by systematically documenting the processes and outcomes of the different capacity building dimensions during all meetings of the stakeholder groups, and supplementing these findings with the participants' perspectives and experiences. Thereby, we try to take into account the dynamics of the process. Capacity is essentially not an existing characteristic, but a process based on the interaction between individuals and groups within the respective setting and is thus subject to continuous fluctuations (15). We aim to grasp these complex interactions and processes by the semi-standardized documentation instrument, which can help record the complex changes expected at different levels and across the timespan [(44), Sauter et al., under review].

Whereas, the overall aim of promoting PA in men 50 plus is set “top-down” by the project funding institution, the stakeholder group participants will ultimately decide via a participatory bottom-up-process on the measures that will be chosen to reach the aim, and will also be invited to engage in further public health issues. Bringing together top-down and bottom-up processes is also referred to as “parallel tracking” (49). This procedure, together with other supporting factors for designing participatory interventions (e.g., early involvement of local partners, strong integration of local politics and involvement of the target group during planning phase) (42) can increase the identification of the stakeholders with the implemented measures and thus the chances of a sustainable effect (10).

However, the complexity of the intervention also may imply different challenges for the research team. By bringing together several relevant stakeholders from a community, existing conflicts of interest are becoming more likely between the actors involved—a challenge that has emerged as an obstacle in numerous participatory interventions (42). In addition, the application of this study design with its variety of data collection methods and ongoing knowledge exchange processes will be very labor-intensive, which may necessitate simplification in order to adapt it to future projects.

## Conclusion

In summary, A4M is designed to expand knowledge and understanding of frameworks and proceedings that are a good fit for capacity building interventions (17). Furthermore, it is expected to foster our understanding of how to consider masculinity in PA promotion interventions. Up to date, systematic approaches regarding such programs can only be found sporadically in Germany (50). The expected research results will provide new insights for health promotion research, research on social work in communities, and sports science. In addition, the results can also be useful for representatives of public health service, and health-related associations by developing recommendations for the design of stakeholder group approaches in local municipalities.

## Ethics and Dissemination

This study was reviewed and approved by the research ethics committees at the University of Bayreuth prior to the start of data collection. Written informed consent was obtained from all participants prior to participating in the study, and participants were reminded that they can withdraw at any time. All spontaneously reported adverse events and other unintended effects of the study will be collected, assessed, and managed by the principal investigators. Adverse events will be reported to the ethics committee and any updates to the study will go through ethics committee approval.

Our dissemination strategy includes a report of the baseline assessment results (pre-survey) on psychosocial correlates of PA as well as the systematic literature review on PA programs to the participants of the stakeholder group. In this way, we strive to advance the scientific basis of knowledge and action of the stakeholder group. Moreover, results from the outcome assessment (changes in psychosocial correlates drawn from the post-survey as well as capacity building process) will be used for a policy report. This report will be shared and discussed with various key organizations, especially focusing on associations on a (supra-) regional level (e.g., Bavarian umbrella sports organization, Bavarian Public Health section, Bavarian Chamber of Physicians, regional ministry of health). The aim of these discussions is to initiate a scaling-up process that transfers the strategies related to the capacity building approach to other communities region- and nationwide. This will foster implementation of participatory strategies in health intervention planning. Additionally, we will present the results to the scientific community to add to the discussion around community capacity building processes (publications in peer-reviewed journals, conference presentations, etc.). Finally, dissemination to the broader public will take place via the homepage of the research consortium (https://www.capital4health.de/en/) as well as public events.

## Author Contributions

HS contributed to study conception and was a major contributor in writing the manuscript. NB-S, JC, ST, and JL contributed to study conception and critical revision of the manuscript. BM contributed to study conception. All authors read and approved the final manuscript.

### Conflict of Interest

The authors declare that the research was conducted in the absence of any commercial or financial relationships that could be construed as a potential conflict of interest.

## Abbreviations

PA: Physical activity
A4M: ACTION for men.
